# Melatonin: a multifaceted regulator of root development, stress responses, and hormonal crosstalk in horticultural crops

**DOI:** 10.3389/fpls.2026.1718959

**Published:** 2026-02-25

**Authors:** Chenglin Liang, Hongpeng Xu, Dingli Li, Chunhui Ma, Yi Lv, Jianlong Liu

**Affiliations:** 1 Haidu College, Qingdao Agricultural University, Laiyang, China; 2 College of Horticulture, Qingdao Agricultural University, Qingdao, China; 3 Weihai Agricultural Science Academy, Weihai, Shandong, China

**Keywords:** antioxidant system, melatonin, nutrient uptake, plant hormones, root development, stress resistance

## Abstract

Melatonin is increasingly recognized as a multifunctional signaling molecule involved in plant growth regulation and stress adaptation. Recent studies have revealed that melatonin plays a pivotal role in shaping root system architecture (RSA) by modulating root growth dynamics, lateral root formation, and root-microbe interactions. This review highlights emerging evidence that melatonin regulates RSA through complex crosstalk with phytohormones, reactive oxygen species, and stress‐responsive signaling pathways, rather than acting solely as a growth regulator. Importantly, accumulating evidence indicates that melatonin functions as an integrative regulator of RSA by coordinating multiple hormone signaling pathways, including auxin, jasmonic acid, ethylene, cytokinins, salicylic acid, and abscisic acid, in a concentration‐ and context‐dependent manner. We further distinguish the regulatory effects of melatonin on root growth and root architectural remodeling and summarize the dose‐dependent actions of melatonin under abiotic stress conditions. Beyond hormonal regulation, melatonin enhances root nutrient acquisition by modulating ion transporters, maintaining ion homeostasis, and optimizing root system architecture, thereby improving nitrogen, phosphorus, potassium, and micronutrient uptake under stress conditions. Emerging evidence also suggests that melatonin may indirectly influence root-microbe interactions by reshaping root physiology, redox status, and hormonal balance, contributing to improved stress resilience. By integrating molecular, physiological, and developmental perspectives, this review provides a conceptual framework for understanding melatonin‐mediated root system plasticity and positions melatonin as an integrative regulator of root system architecture that links hormonal crosstalk, nutrient acquisition, and stress adaptation, offering insights into its potential applications in crop stress resilience improvement.

## 1 Introduction

During the growth and development stages of horticultural crops, there are often stressful environments that pose significant challenges to survival. This adversity is a major limiting factor for crop yield, frequently leading to reduced yields in many cases (Gong et al., 2017; Ni et al., 2018; Kashyap et al., 2021; Wei et al., 2021). To cope with these stress conditions, plant organs undergo a series of physiological changes to alleviate the negative effects of adverse environments (Gong et al., 2023; Zheng et al., 2023).

The exogenous application of plant growth regulators has been proven to mitigate the adverse effects of environmental stress on crops through various means (Guo et al., 2023). As the environmentally friendly agricultural chemical, melatonin is widely used in production and holds tremendous application potential in the horticultural market, addressing the challenges faced by the agricultural industry (Sun et al., 2021; Gao et al., 2022; Wang et al., 2022a).

Melatonin (N-acetyl-5-methoxytryptamine) is an indoleamine molecule widely present in biological organisms. In animals, it participates in circadian rhythm, sleep, immune regulation, and other physiological processes (Jan et al., 2009; Hardeland et al., 2012; Carrillo-Vico et al., 2013). It also acts as a free radical scavenger and antioxidant (Tan et al., 2012; Wang et al., 2012; Gong et al., 2017; Wei et al., 2018). In 1995, Dubbels et al. first discovered melatonin in higher plants (Dubbels et al., 1995), and subsequent studies confirmed its widespread distribution in roots, stems, leaves, fruits, and seeds.

In plants, melatonin is typically synthesized in chloroplasts and mitochondria (Zeng et al., 2022a). The synthesis process involves a series of enzymatic reactions starting with tryptophan: first, tryptophan decarboxylase (TDC) catalyzes the decarboxylation of tryptophan to form tryptamine. Subsequently, tryptamine is hydroxylated to serotonin (5-hydroxytryptamine, 5-HT) under the catalysis of tryptamine 5-hydroxylase (T5H). The subsequent conversion of serotonin to melatonin requires two sequential enzymatic steps, mediated by serotonin N-acetyltransferase (SNAT) and N-acetylserotonin O-methyltransferase (ASMT; also referred to as caffeic acid O-methyltransferase, COMT) (Rehaman et al., 2021; Kolupaev et al., 2024; Wang et al., 2024). Notably, the order of these enzymatic transformations can vary under different environmental conditions (Zeng et al., 2022a). Under standard growth conditions, SNAT first catalyzes the acetylation of serotonin to produce N-acetyl 5-hydroxytryptamine (aHT), which is then O-methylated by ASMT/COMT to generate melatonin (Ye et al., 2019). In contrast, abiotic stress induces the expression of distinct ASMT/COMT isoforms, leading to an altered reaction order: serotonin is first O-methylated to 5-methoxytryptamine (5-MT), and 5-MT is subsequently acetylated by SNAT to form melatonin (Tan and Reiter, 2020).

Melatonin regulates multiple plant processes, including explant, root, and shoot growth, seed germination, root development, and leaf senescence delay (Byeon and Back, 2014; Tijero et al., 2019; Dai et al., 2020; Khan et al., 2020; Zhao et al., 2021a). It is also involved in plant stress responses, protecting plants from adverse environmental conditions (Qi et al., 2018; Zhou et al., 2022; Kwon et al., 2025). Notably, exogenous melatonin plays a crucial role in root organogenesis and lateral root development in horticultural plants (Park and Back, 2012; Zhang et al., 2014; Javeed et al., 2021), and can mitigate environmental impacts on root growth by regulating physiological processes (Bajwa et al., 2014; Ren et al., 2022a, b; Wang et al., 2025).

Melatonin’s amphiphilicity and stability enable it to be transported from roots to aerial tissues via the apoplast, serving as a long-distance messenger to induce systemic disease resistance (Erland et al., 2019; Yoon et al., 2019; Song et al., 2022). Its function in enhancing root vitality is closely linked to high crop yields, making it a promising regulator for sustainable horticultural production. Rather than functioning as a single growth regulator or hormone analog, melatonin emerges as an integrative regulator of root system architecture, coordinating multiple signaling networks in a context- and concentration-dependent manner.

## 2 Effects of melatonin on root growth and root system architecture

**Figure 1:**
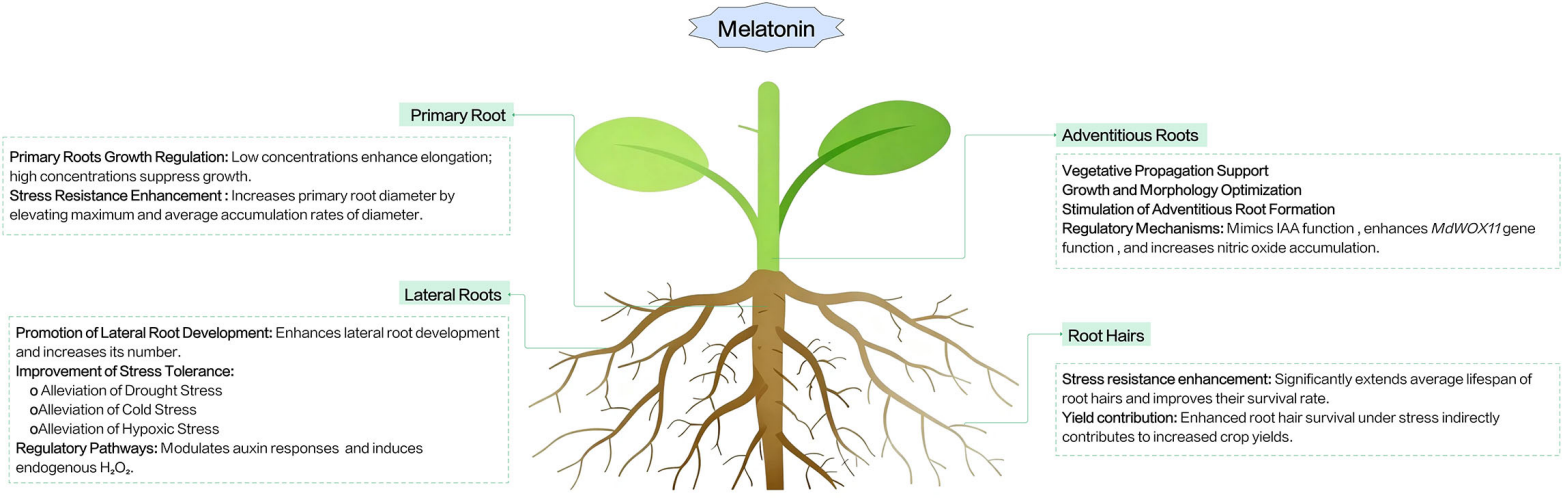
Conceptual model of melatonin’s effects on plant root systems. This model summarizes generalized effects, which are concentration-dependent and species-specific. Low concentrations of melatonin promote primary root elongation, while high concentrations inhibit it by reducing IAA synthesis and transport gene transcription levels. Exogenous melatonin application extends medium - diameter lateral root lifespan, increases survival rate, and reduces death proportion. Transgenic plants overexpressing melatonin produce more lateral roots than wild - type ones. Melatonin increases lateral root density and relieves stress - induced growth inhibition. It modulates auxin responses (promoting root primordium morphogenesis with IAA) and induces endogenous H_2_O_2_ to trigger lateral root formation. Moreover, it enhances growth by increasing adventitious root number and optimizing root morphology, and promotes adventitious root formation by enhancing root development - related gene expression.

Melatonin plays a crucial role in regulating plant root growth and shaping root system architecture (RSA), affecting multiple root components, including primary roots, lateral roots, adventitious roots, and root hairs (**Figure 1**) (Erland et al., 2019). Root system architecture emerges from the coordinated development of different root types, which are initiated at distinct developmental stages and regulated by both endogenous signals and environmental cues (Yalamanchili et al., 2024).

### 2.1 Regulation of primary root growth by melatonin

The primary root is established during embryogenesis and forms the central axis of the root system, largely determining rooting depth and the plant’s ability to access deep soil water resources (Liu and von Wirén, 2022). Increased primary root diameter and elongation capacity enhance penetration into compact or dry soils, thereby improving drought resistance (Clark et al., 2008; Bengough et al., 2011). Zhu et al. (2023) demonstrated that melatonin significantly increased primary root diameter in cotton under drought stress by elevating both the maximum and average accumulation rates of root diameter (Zhu et al., 2023).

The regulatory effects of melatonin on primary root growth are strongly concentration-dependent. Low concentrations generally promote primary root elongation, whereas higher concentrations inhibit growth, primarily through the suppression of indole-3-acetic acid (IAA) biosynthesis and transport gene expression (Wang et al., 2016; Yang et al., 2021b). Hernandez-Ruiz et al. (2005) reported that low melatonin concentrations stimulated primary root growth in wheat (*Triticum aestivum* L.). However, inhibitory effects were observed in monocotyledonous species such as *Phalaris canariensis* L. and *Avena sativa* L., highlighting the species-specific nature of melatonin responses (Hernandez-Ruiz et al., 2005).

### 2.2 Melatonin-mediated regulation of lateral roots and root hairs

Lateral roots are essential for increasing root surface area, mechanical stability, and nutrient and water uptake efficiency (Hu et al., 2020). Environmental stresses often accelerate lateral root senescence and shorten their lifespan (Liu et al., 2023b; Shao et al., 2023). Under drought stress, cotton lateral roots exhibit reduced longevity and increased mortality. However, melatonin application significantly enhances lateral root survival by extending the lifespan of medium-diameter lateral roots (Zhu et al., 2023).

Exogenous melatonin has been widely reported to promote lateral root initiation and development across multiple species (Arnao and Hernandez-Ruiz, 2007; Chen et al., 2019). Under cold stress, transgenic rice seedlings overexpressing melatonin produced more lateral roots than wild-type plants (Kang et al., 2010), while under hypoxic stress, melatonin increased lateral root density and alleviated stress-induced inhibition in rice seedlings (Liu et al., 2023a). Mechanistically, melatonin regulates lateral root formation by modulating auxin signaling pathways (Liang et al., 2017). Following indole-3-acetic acid (IAA) application, melatonin treatment in lupine enhanced root primordia morphogenesis and increased both lateral and adventitious root numbers (Arnao and Hernandez-Ruiz, 2007).

In addition to auxin-related regulation, melatonin-mediated lateral root development is closely associated with reactive oxygen species (ROS) signaling. In alfalfa seedlings, melatonin induces lateral root formation by stimulating endogenous H_2_O_2_ production, and overexpression of the melatonin biosynthesis gene *MsSNAT* in *Arabidopsis thaliana* produces similar phenotypes (Chen et al., 2018). Comparable promotive effects of melatonin on lateral root growth have also been reported in tomato and cucumber (Zhang et al., 2014; Chen et al., 2019). In *Arabidopsis thaliana*, melatonin treatment resulted in a threefold increase in lateral root emergence (Pelagio-Flores et al., 2012).

Root hairs are another critical component of RSA, enhancing the root–soil interface and facilitating nutrient and water uptake under adverse environmental conditions (Brown et al., 2012; Jones and Dolan, 2012; Marin et al., 2021; Rongsawat et al., 2021). Drought stress significantly reduces root hair lifespan in cotton (Xiao et al., 2020), whereas melatonin application markedly prolongs root hair longevity, improves survival under drought stress, and ultimately contributes to higher cotton yields (Zhu et al., 2023).

### 2.3 Regulation of adventitious root formation by melatonin

Adventitious roots originate from non-root tissues such as hypocotyls, stems, or leaves and are regulated by complex interactions between environmental signals and phytohormones (Li et al., 2009; Gonin et al., 2019). Auxin, cytokinin, jasmonic acid, salicylic acid, and ethylene all participate in adventitious root formation (Zhi-Guo et al., 2012; Gonin et al., 2019; Pan et al., 2021; Li et al., 2023). Adventitious rooting is a key process in vegetative propagation of economically important crops.

Melatonin treatment significantly enhances adventitious root formation and rooting success (Wang et al., 2023). In grapevines, exogenous melatonin increased adventitious root number, improved root morphology, and enhanced root growth under salt stress (Yu et al., 2022). Similar promotive effects have been observed in cucumber, where melatonin stimulated adventitious root formation and improved RSA by upregulating genes associated with root development (Zhang et al., 2014). Physiologically, melatonin exhibits auxin-like activity, and Wen et al. (2016) demonstrated that 50 μM melatonin was optimal for stimulating adventitious root formation in tomato plants (Wen et al., 2016).

At the molecular level, melatonin promotes adventitious root development by regulating auxin metabolism and signaling. In apple, melatonin enhances adventitious rooting by activating *MdWOX11*, a key regulator of auxin-responsive root development (Mao et al., 2020). Additionally, melatonin-induced accumulation of nitric oxide contributes to adventitious root regeneration. Melatonin also promotes rooting of young branches and cuttings, playing an important role in vegetative propagation of fruit tree rootstocks such as sweet cherry and pomegranate (Sarropoulou et al., 2012b; Sarrou et al., 2014).

### 2.4 Concentration-dependent effects of melatonin on root growth and architecture

The regulatory role of melatonin in root growth and RSA is characterized by a distinct concentration-dependent pattern (Sarropoulou et al., 2012a; Mukherjee et al., 2014; Chen et al., 2019; Wang et al., 2021a; Silva et al., 2025). Generally, exogenous melatonin at low concentrations facilitates root elongation, lateral root initiation, and overall root system development, while high concentrations tend to suppress root growth processes (Arnao and Hernández-Ruiz, 2017; Yang et al., 2021b).

This concentration-dependent regulatory pattern has been validated across multiple plant species. For instance, melatonin in the range of 0.01-1 μM promotes root growth in wheat, whereas the same concentration range exerts an inhibitory effect on oat root elongation (Hernandez-Ruiz et al., 2005). In maize, 10 μM melatonin serves as a growth stimulant for roots (Zhao et al., 2015), while 100 μM melatonin inhibits root growth in Canary grass (Hernandez-Ruiz et al., 2005). For sweet cherry rootstocks, the optimal concentration for promoting rooting lies between 0.1-1 μM, and a 10 μM concentration leads to significant inhibition of root development (Sarropoulou et al., 2012b). In lupine, melatonin positively regulates root growth within the concentration window of 1×10–^8^ to 1×10–^5^ M, with 10 μM identified as the optimal concentration, whereas 100 μM induces growth suppression (Hernandez-Ruiz et al., 2005). In rice, exogenous melatonin at 10, 20, and 50 μM enhances lateral root growth (Liang et al., 2017), while 100 μM melatonin improves root system development in kiwifruit under drought stress (Liang et al., 2019).

Furthermore, under abiotic stress conditions, the concentration-dependent effects of melatonin become more intricate, being closely modulated by stress types and plant genotypes (Wang et al., 2024). For example, 300 μM melatonin promotes germination, a prerequisite for root establishment, in two wheat varieties but inhibits this process in another under salt stress, which reflects genotype-specific differences in dose responsiveness (Zhang et al., 2022).

Collectively, these findings indicate that the promotive effects of melatonin on root growth and RSA operate within a relatively narrow concentration window and are highly species- and context-dependent. Therefore, careful optimization of melatonin dosage is essential for exploiting its potential in improving root system development and crop stress resilience.

## 3 The relationship between melatonin and different hormones in promoting root system changes

**Figure 2:**
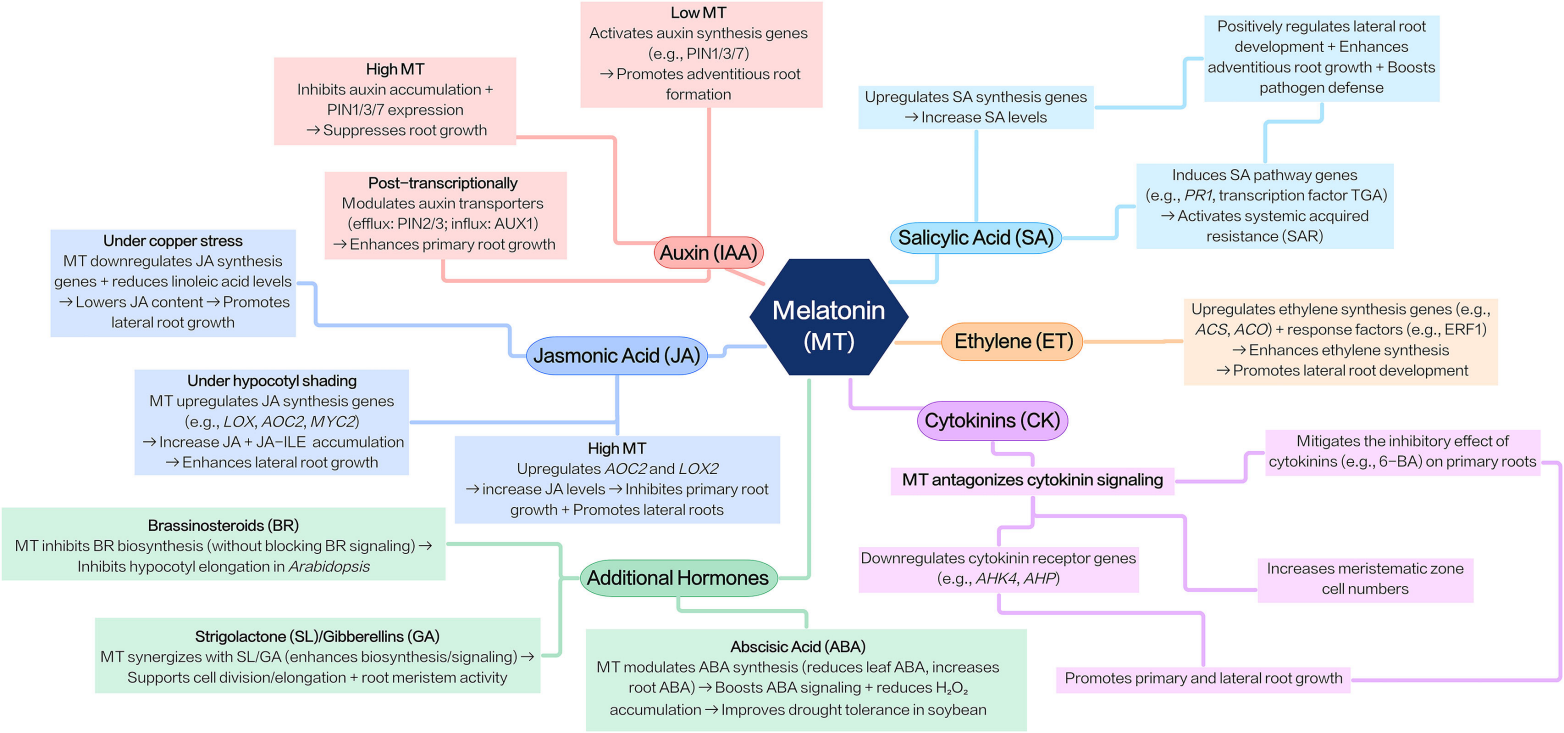
Melatonin-hormone interactions in plant root regulation. Melatonin regulates plant root growth and development by interacting with multiple endogenous hormones (altering their levels, related gene expression, or signaling pathways), with effects that are concentration-dependent. Its core role is to modulate hormone metabolism rather than control roots independently, and most studies confirm it promotes root growth (e.g., lateral/adventitious roots).

The growth and development of higher plants are inseparable from the regulation of plant hormone signals. Plant hormones can regulate important plant biological processes such as growth, development, and environmental responses (Zhu, 2002; Giovannoni, 2004; Vanneste and Friml, 2009; Wang et al., 2022b). Melatonin serves as an important regulator of the metabolism of endogenous plant hormones (such as auxin (IAA), ethylene (ET), jasmonic acid (JA), salicylic acid (SA), etc.) (Arnao and Hernández-Ruiz, 2018). Melatonin treatment can regulate the content of endogenous hormones and the expression of related synthesis genes, thereby stimulating the growth and development of plant roots and promoting the production of new lateral roots and adventitious roots (**Figure 2**) (Lee et al., 2015; Mao et al., 2020; Yang et al., 2021a).

### 3.1 The relationship between melatonin and auxin in promoting changes in the root system

The growth and development of plant roots are dependent on the regulatory role of auxin. Auxin can promote root growth and is an important factor for plant organ regeneration (Xie et al., 2000; Overvoorde et al., 2010). Melatonin and auxin both belong to the class of indoleamine small molecules and share tryptophan as a synthetic substrate in biosynthesis (Murch et al., 2000; Tan et al., 2016). Melatonin regulates plant root growth by influencing endogenous auxin levels and inducing the expression of auxin synthesis genes (Chen et al., 2009; Arnao and Hernández-Ruiz, 2017; Lv et al., 2021). Melatonin can promote root development in apples, tomatoes, rice, and other plants by regulating the synthesis of endogenous auxin (Wen et al., 2016; Liang et al., 2017; Mao et al., 2020; Yang et al., 2021b). Low concentrations of exogenous melatonin can activate the expression of auxin synthesis genes *PIN1/3/7*, thereby promoting the formation of adventitious roots in tomatoes (Wen et al., 2016). However, in *Arabidopsis thaliana*, high concentration of melatonin inhibited the accumulation of auxin and inhibited the expression of *PIN1/3/7* in the auxin signaling process to inhibit root growth (Wang et al., 2016). At the post-transcriptional level, melatonin promotes primary root growth by modulating the expression and distribution of auxin efflux transporters PIN2/3 and influx transporter AUX1, possibly through miRNA-mediated regulation (e.g., downregulating *miR167* that targets PIN2 mRNA) or protein phosphorylation (Wang et al., 2023; Yang et al., 2021b). Melatonin promotes root growth in wild type *Arabidopsis*. However, melatonin only partially rescues root growth inhibition after mutation of auxin efflux transporters (*pin3/7* and *pin4/7* mutants) or in the presence of auxin pathway inhibitors at low concentrations. Notably, melatonin has no effect on promoting root growth in the yucQ mutant or in the presence of high concentrations of auxin pathway inhibitors (Yang et al., 2021b). Therefore, it is speculated that melatonin may not be an auxin-like hormone but rather a plant hormone that regulates plant root growth in an IAA-dependent manner or in parallel with IAA (Koyama et al., 2013). However, there have also been reports that present different perspectives, no changes in auxin-responsive promoters (DR5:GUS) or heat shock promoters (HS::AXR3NT)-GUS were observed in transgenic *Arabidopsis* seedlings treated with melatonin (Pelagio-Flores et al., 2012). These studies suggested that melatonin promoted rooting in an independent manner, not dependent on auxin. Therefore, further in-depth research is needed to further determine the regulatory mechanism of melatonin in the rooting process.

### 3.2 The relationship between melatonin and jasmonic acid in promoting changes in the root system

Jasmonic acid plays a significant role in regulating plant root development. It inhibits root development by suppressing cell division and root cell elongation (Mandal et al., 2025), and also suppresses the growth and development of primary roots in *Arabidopsis* (Chen et al., 2011). Conversely, melatonin can downregulate the expression of jasmonic acid-related synthesis genes and reduce the level of linoleic acid, thereby decreasing jasmonic acid levels and promoting the development of lateral roots in melon roots under copper stress (Hu et al., 2020). In terms of plant lateral root regulation, jasmonic acid acts as a hormonal signal that stimulates rooting and can promote the formation of lateral roots in peas, tobacco, *Arabidopsis*, and petunias (Fattorini et al., 2009; Lischweski et al., 2015; Rasmussen et al., 2015). Under conditions of shading the hypocotyls of plants, melatonin can upregulate the expression of the jasmonic acid synthesis gene *LOX*, promoting the accumulation of JA and jasmonic acid-L-isoleucine (JA-ILE). Simultaneously, the upregulation of the jasmonic acid synthesis gene *MYC2* under the induction of exogenous melatonin can further activate the expression of JA response genes, thereby promoting the growth of plant lateral roots (Wang et al., 2022b). High concentration of melatonin promotes the expression of *AOC2* and *LOX2*, increases the content of myricic acid to inhibit the growth of main roots and promote the development of lateral roots (Yang et al., 2021a).

### 3.3 The relationship between melatonin and ethylene in promoting changes in root systems

The accumulation of ethylene is necessary for the generation of adventitious root primordia and the formation of aerenchyma cells in plants (Yamauchi et al., 2014, 2016). During plant root development, ethylene interacts with auxin, ABA, and gibberellin, regulating the formation of lateral roots in horticultural crops such as rice and tomatoes (Steffens et al., 2006; Vidoz et al., 2010). When plants encounter waterlogging stress, ethylene is produced in the roots, inducing the formation of root aerenchyma under hypoxic conditions and promoting the development of adventitious roots. Ethylene synthesis-related genes *ACO* and *ACS* are significantly upregulated during lateral root formation in petunias (Lischweski et al., 2015). Melatonin can promote the expression of ethylene synthesis-related genes and enhance the development of lateral roots in plants. In *Arabidopsis*, melatonin regulates the expression of key ethylene synthase genes *ACS* and *ACO*, thereby promoting the formation of lateral roots (Yang et al., 2021a). Ethylene response factors (ERFs) are common mediators of root development, and ERF1 is significantly induced under shading conditions in the hypocotyls of plants treated with melatonin. In cucumber seedlings treated with exogenous melatonin, ethylene synthesis and its response factor genes were significantly expressed, indicating that melatonin may promote lateral root development by enhancing ethylene synthesis (Wang et al., 2022b).

### 3.4 The relationship between melatonin and cytokinins in promoting changes in the root system

Cytokinins, as crucial plant growth regulators, play a significant role in regulating plant cell division and root development (Werner et al., 2001; Sakakibara, 2006). Cytokinins control root cell differentiation and determine the size of plant root meristems (Dello Ioio et al., 2007). Cytokinins have a negative regulatory role in regulating the elongation of primary roots and the growth of lateral roots in plants (Zubo et al., 2017). Exogenous melatonin can regulate the cytokinin response pathway, thereby increasing the number of root cells in the meristematic zone of plants and participating in the regulation of the growth of primary roots. In *Arabidopsis*, 10 nM exogenous melatonin can inhibit the negative regulatory effect of 6-BA on the elongation of primary roots, while downregulating the expression of cytokinin receptors AHK4 and AHP to inhibit endogenous cytokinin responses (Wang et al., 2023). This indicates that melatonin can inhibit cytokinin signaling and promote the growth of primary and lateral roots.

### 3.5 The relationship between melatonin and salicylic acid in promoting changes in the root system

Salicylic acid affects plant seed germination, lateral root development, root elongation, and defense against pathogens (Pasternak et al., 2019; Dong et al., 2020). Salicylic acid enhances the growth of adventitious roots, promotes lignification of the cell wall and closure of plasmodesmata, thereby directly preventing the invasion of pathogens into plant roots and promoting normal growth of plants under biotic stress (Bagautdinova et al., 2022). The expression of the *PR1* gene is considered a marker for activating SA-mediated systemic acquired resistance in plants. Exogenous melatonin can induce the expression of *PR1* and transcription factor TGA, mediating plant systemic acquired resistance, resisting pathogen infection during lateral root formation, and promoting lateral root growth (Wang et al., 2022b). Salicylic acid can synergistically regulate plant lateral root development with auxin. Under flooding conditions, exogenous SA can promote the formation of lateral roots in wheat (Koramutla et al., 2022). The number of lateral roots produced in *Arabidopsis* mutants lacking SA synthesis is significantly lower than that of normal *Arabidopsis* plants (Gutierrez et al., 2012). This indicates that salicylic acid is a positive regulatory factor for plant lateral root development. Exogenous melatonin can upregulate the expression of salicylic acid synthesis genes and promote the increase of SA levels (Lee et al., 2015). This suggests that the development of plant lateral roots induced by exogenous melatonin may be related to the increase in salicylic acid levels.

### 3.6 The relationship between melatonin and other hormones in promoting changes in the root system

In addition to the aforementioned hormones, melatonin can also regulate hormones such as brassinosteroids (BR) and gibberellins (GA) to mediate root growth (Yang et al., 2021a; Tian et al., 2024). Accumulating evidence indicates that melatonin functions as a central modulator of root system architecture by integrating multiple phytohormone pathways rather than acting through a single linear signaling route. Studies have shown that melatonin promoted adventitious root formation in cucumber seedlings by regulating the expressions of genes related to hormone synthesis, signaling and cell wall formation, as well as by increasing the contents of IAA, CK, JA, SA and ABA (Wang et al., 2022b). Melatonin potentially improved drought tolerance of soybean through the regulation of ABA and aquaporin gene expression, increasing photosynthetic efficiency as well as enhancing water uptake efficiency. Specifically, melatonin prevented ABA synthesis in leaves and increased endogenous ABA content in roots, thereby boosting ABA signaling and generating less H_2_O_2_ accumulation in plant under osmotic stress (Jahan et al., 2023). In addition, melatonin inhibited BR biosynthesis but did not block BR signaling in the inhibition of hypocotyl elongation in *Arabidopsis* (Xiong et al., 2019). In contrast, melatonin synergized with growth-promoting hormones, including strigolactone (SL) and gibberellins (GAs), by enhancing their biosynthesis and signaling capacity, which supported cell division, cell elongation, and root meristem activity (Yang et al., 2021a; Tian et al., 2024). Through these coordinated antagonistic and synergistic interactions, melatonin fine-tunes hormonal homeostasis to optimize root development and stress adaptation. Nevertheless, the molecular nodes linking melatonin signaling to hormone biosynthesis, transport, and transcriptional regulation remain incompletely understood, underscoring the need for integrative genetic, molecular, and biochemical studies to clarify whether melatonin acts upstream of, in parallel with, or downstream of canonical phytohormone signaling networks. Elucidating the crosstalk between melatonin and hormones will not only enhance our comprehension of root developmental regulation but also offer a theoretical basis for enhancing crop resilience and optimizing the root system in agricultural and horticultural production.

### 3.7 Integrative view of melatonin-centered hormonal crosstalk in RSA regulation

Although melatonin interacts with multiple phytohormones to regulate root system architecture, these interactions are not isolated but form an integrated regulatory network (Arnao and Hernández-Ruiz, 2018; Gao et al., 2022). Current evidence suggests that melatonin functions as a central coordinator rather than a linear upstream or downstream signal (Yang et al., 2021a; Wang et al., 2023). At low concentrations, melatonin preferentially enhances auxin biosynthesis and transport, thereby promoting root meristem activity and lateral/adventitious root initiation (Liang et al., 2017; Mao et al., 2020). Under stress conditions, melatonin simultaneously attenuates inhibitory hormonal signals such as cytokinins and jasmonic acid while enhancing adaptive signals including ethylene and salicylic acid, enabling flexible root system remodeling (Chen et al., 2025; Hu et al., 2020; Arnao and Hernández-Ruiz, 2018).

This multilayered hormonal coordination allows melatonin to prioritize root growth, stress defense, or developmental plasticity depending on environmental context (Yang et al., 2021b; Gao et al., 2022). Such a hierarchical and context-dependent regulation may explain the concentration-dependent and species-specific effects of melatonin on root architecture (Hernández-Ruiz et al., 2005; Sarropoulou et al., 2012a; Silva et al., 2025). Future studies integrating hormone mutants, transcriptomics, and spatial hormone profiling will be essential to decipher how melatonin dynamically orchestrates these signaling pathways (Bian et al., 2021; Cano et al., 2024).

## 4 Melatonin regulates nutrient uptake in plant roots

Melatonin-regulated nutrient acquisition constitutes a critical component of root stress responses and adaptive root system function. This process involves multiple molecular mechanisms, including the modulation of ion transporters, maintenance of ion homeostasis, and optimization of root architecture, which collectively promote the absorption of macronutrients (e.g., nitrogen, phosphorus, potassium) and micronutrients (e.g., iron, zinc, calcium) (Arnao and Hernández-Ruiz, 2015; Nawaz et al., 2018).

### 4.1 Regulation of nitrogen (N) uptake

Nitrogen is a fundamental nutrient for plant growth, and melatonin significantly facilitates root N uptake under both normal and stress conditions. In apple plants subjected to moderate drought stress, exogenous melatonin application upregulated the expression levels of uptake genes (*AMT1;2*, *AMT1;5*, *AMT1;6*, *AMT2;1*, *NRT1;1*, *NRT2;4*, *NRT2;5*, *NRT2;7*), thereby enhancing the uptake and accumulation of δ^15^N-labeled urea (Liang et al., 2018). This effect was associated with the restoration of N-metabolism enzyme activities (nitrate reductase, nitrite reductase, glutamine synthetase, glutamate synthase), which are typically suppressed by drought stress (Huang et al., 2018a, b). Similarly, in peach seedlings, 150 μM melatonin treatment increased N contents in roots and shoots by 15.16% and 12.43%, respectively, compared to the control, by improving soil nutrient availability and root absorption capacity (Liu et al., 2022). The synergistic application of melatonin and IAA further enhanced N uptake in salt-stressed maize, with the Pearl cultivar showing a notable increase in leaf N content under salinity stress (Ali et al., 2025), indicating that melatonin may interact with phytohormones to reinforce N acquisition.

### 4.2 Promotion of phosphorus (P), potassium (K), and micronutrient uptake

Melatonin also plays a critical role in regulating the uptake of P, K, and essential micronutrients. Under drought stress, melatonin application increased P concentrations in apple roots by 13.8% and enhanced P uptake flux by 29.2% compared to drought-stressed plants without melatonin (Liang et al., 2018). In peach seedlings, the optimal melatonin concentration (150 μM) maximized P and K contents in shoots, with K uptake in roots increased by 14.16% (Liu et al., 2022). For micronutrients, melatonin pretreatment alleviated nickel (Ni)-induced nutrient deficiency in tomato seedlings by increasing root Fe, Mn, Ca, and Mg contents by 27.57-44.48% compared to Ni-stressed plants (Altaf et al., 2021). In salt-stressed maize, the combined application of melatonin and IAA significantly improved Zn and Fe uptake, which are crucial for maintaining photosynthetic efficiency and antioxidant capacity (Ali et al., 2025). Additionally, melatonin reduced Na^+^ and Cl^-^ accumulation in maize roots and leaves, maintaining ion homeostasis by balancing the uptake of beneficial ions (e.g., Ca^2+^, K^+^) against toxic ions (Ali et al., 2025), a mechanism also observed in tomato under Ni stress (Altaf et al., 2021).

### 4.3 Molecular mechanisms underlying melatonin-mediated nutrient uptake

The regulatory effects of melatonin on root nutrient uptake are governed by multiple molecular pathways. First, melatonin modulates the expression of nutrient transporter genes, as evidenced by the upregulation of AMT and NRT families in apple (Liang et al., 2018) and enhanced K^+^ transporter gene expression in Malus (Li et al., 2016). Second, melatonin maintains ion homeostasis through NO-dependent pathways, as reported in previous studies (Siddiqui et al., 2019), which activate H^+^ pumps to generate proton gradients essential for nutrient symport (Zhang et al., 2017). For example, melatonin-induced H^+^-ATPase activity in roots facilitates the absorption of cations (e.g., K^+^, Ca^2+^) and anions (e.g., NO_3_^-^) (Arnao and Hernández-Ruiz, 2014). Third, melatonin optimizes root architecture to improve nutrient foraging, such as increasing root length, surface area, and root tips in peach (Liu et al., 2022) and tomato (Altaf et al., 2021), which enhances the contact area between roots and soil nutrients. Furthermore, melatonin reduces oxidative damage to root cells by enhancing antioxidant enzyme activity (SOD, CAT, APX), preserving root cell membrane integrity and nutrient transport function under stress (Altaf et al., 2021; Ali et al., 2025).

## 5 Melatonin enhances plant resistance through root systems

Melatonin, as a small molecule capable of scavenging reactive oxygen species, has been proven to participate in various stress responses to enhance plant resistance. In this process, melatonin’s involvement in the establishment of root architecture under adverse environmental stresses is also one of the important pathways to improve plant resistance. Due to the plasticity limitations of plants themselves, the impact of environmental stresses such as drought, salt stress, and cold damage on crops varies depending on the severity, timing, and duration of the stress (Farooq et al., 2009; Hu and Xiong, 2014).

### 5.1 Drought stress

Drought stress hinders plant growth by reducing water absorption in plant cells and has adverse effects on many physiological and biochemical reactions (Khan et al., 2025). When exposed to such unfavorable conditions, plants exhibit abnormal development due to inhibited cell proliferation and expansion (Skirycz and Inze, 2010). Exogenous melatonin can alleviate drought stress in most plants, positively affect plant growth, and increase crop yield (Wang et al., 2013; Zhang et al., 2013; Arnao and Hernández-Ruiz, 2014; Meng et al., 2014; Arnao and Hernández-Ruiz, 2015; Liu et al., 2015; Jahan et al., 2020). In cotton root growth, the root length under drought stress is only one-quarter of that in the control treatment, while the application of exogenous melatonin can effectively alleviate this situation. Altaf et al. found that the application of melatonin can increase the root length, root volume, and root surface area of tomatoes (Altaf et al., 2021).

### 5.2 Waterlogging stress

Besides drought stress, waterlogging stress can also damage plant roots. Excessive waterlogging hinders air exchange between the soil and the atmosphere, thus inhibiting root respiration and reducing root vitality. High concentrations of ROS caused by waterlogging stress can easily cause oxidative damage to plant roots (Colmer and Flowers, 2008; Sauter, 2013; Yamauchi et al., 2018; Yildirim and Demir, 2022). As an antioxidant and free radical scavenger, exogenous melatonin can effectively enhance the antioxidant enzyme activity of horticultural crop roots under waterlogging stress, reduce the accumulation of reactive oxygen species in the roots, and alleviate the oxidative damage caused by waterlogging stress (Gu et al., 2021). Exogenous melatonin can increase the O^2-^ content in rice roots under hypoxia stress, promote the length of primary roots and lateral roots, and reduce oxidative damage (Liu et al., 2023b). During flooding, melatonin can enhance the activities of SOD and POD in peaches, reduce lipid peroxidation and ethylene content, indicating that melatonin can act as an antioxidant under flooding stress (Gu et al., 2021). In addition to scavenging excessive reactive oxygen species, melatonin can also enhance the synthesis of proline and γ-aminobutyrate in kiwi roots, promote the flood resistance of plants, and slow down the senescence rate of roots under waterlogging stress (Huo et al., 2022).

### 5.3 Salt stress

Salt stress can alter the phenotype of plant roots and increase reactive oxygen species (ROS) concentration, thereby inhibiting the normal development of root-related phenotypes such as root length and root surface area (Jbir et al., 2001; Morton et al., 2019). Exogenous melatonin can alleviate this inhibitory effect through multiple pathways, and its regulatory mechanism not only involves its well-documented antioxidant capacity, but also enhances plant salt stress tolerance by fine-tuning redox signaling and ion homeostasis in roots (Michard and Simon, 2020). Unlike simple ROS scavengers, melatonin can preferentially scavenge highly toxic hydroxyl radicals (‧OH) while preserving hydrogen peroxide (H_2_O_2_)-mediated signaling, allowing stress signaling to proceed without causing excessive cellular damage (Michard and Simon, 2020). In addition, it can increase the activity of antioxidant enzymes and enhance plant antioxidant capacity, which has been confirmed in studies on sorghum seedlings. Exogenous melatonin can increase the antioxidant capacity and root activity of sorghum seedlings, alleviating the inhibitory effect of salt stress on their growth (Nie et al., 2023).

In terms of ion homeostasis regulation, melatonin is crucial for maintaining cytosolic K^+^/Na^+^ balance in roots, which is also a key determinant of salt tolerance in glycophytic plants. Under saline conditions, Na^+^ influx depolarizes the plasma membrane and induces massive K^+^ efflux, leading to ionic imbalance and growth inhibition. Melatonin can significantly reduce salt-induced K^+^ leakage from roots by regulating NADPH oxidase (RBOH)-dependent ROS production and downstream Ca^2+^ signaling pathways, which in turn regulate the activity and expression of K^+^ transporters and channels. This regulatory effect is not a mitigation in the late stage of stress, but an early regulation of the root ion transport system during stress perception. At the same time, melatonin can also promote the accumulation of essential nutrient ions such as Ca^2+^ and Mg^2+^, and reduce the accumulation of toxic ions such as Cl^-^ and Na^+^ (Michard and Simon, 2020). For example, studies on apples have shown that it can enhance salt tolerance by regulating ion homeostasis, and Kumari et al. also confirmed that exogenous melatonin can enhance plant salt stress tolerance by regulating root morphology and ion absorption (Kumari et al., 2023). In addition, melatonin can promote root activity, regulate root structure and nutrient absorption, thereby further alleviating the inhibitory effect of salt stress. For instance, under salt stress, exogenous melatonin can increase the root length, volume and surface area of tomatoes (Zeng et al., 2018; Siddiqui et al., 2019).

### 5.4 Heavy metal stress or non-metal stress

The lack or excess of metals or non-metals in the soil is also one of the important reasons for crop yield reduction (Ouni et al., 2024). Nitrogen is an essential macronutrient for plant growth and development, and plant growth can be inhibited when nitrogen is deficient. Studies have shown that NO^3-^ can stimulate the elongation of lateral roots in plants, and exogenous melatonin can increase the intake of NO^3-^ in plants, thereby promoting the development of plant roots and facilitating the absorption of N elements by plant roots. High concentrations of copper ions in the soil can inhibit the growth and development of *Arabidopsis* roots and stems (Kolbert et al., 2012), while melatonin can improve plant tolerance to stress by promoting plant root development and reducing oxidative damage (Hu et al., 2020). Arsenic is a harmful non-metal in the environment and poses potential hazards to plant growth and development (Siddiqui et al., 2020; Singh et al., 2021). The most common form of arsenic in flooded paddy soil is arsenite (Zhao et al., 2013). Excessive arsenite can easily cause damage to plant roots (Zeeshan et al., 2023). Under arsenic stress, exogenous melatonin can increase the activities of SOD, APX, and POD in tea plants (Li et al., 2021), enhance the activities of SOD, APX, POD, and CAT in rosemary, and promote root growth (Farouk and Al-Amri, 2019). Melatonin can also alleviate the growth inhibition of *Arabidopsis* caused by excessive copper and cadmium, as well as the inhibition of tomato caused by aluminum stress (Maksymiec et al., 2005; Bali et al., 2019). Therefore, melatonin is often used to improve plant tolerance to heavy metal stress or non-metal stress (Zhang et al., 2015; Siddiqui et al., 2020; Zeng et al., 2022a).

### 5.5 Regulation of stress-responsive gene networks in roots by melatonin

Beyond its antioxidant capacity, melatonin plays a pivotal role in regulating stress-responsive gene networks in plant roots (Zhao et al., 2019; Bian et al., 2021). Transcriptomic analyses of melatonin-treated plants under abiotic stress have consistently shown differential expression of multiple transcription factor families, including WRKY, AP2/ERF, MYB, NAC, and bZIP, which are central regulators of stress signaling and root adaptive responses (Zhao et al., 2021b; Li et al., 2022). For example, in melatonin-treated maize roots under drought, genes encoding WRKY, AP2/ERF, MYB, NAC and bZIP transcription factors were upregulated, indicating enhanced activation of stress signaling and downstream defense pathways (Zhao et al., 2021b). Similarly, in alfalfa roots, melatonin induced transcriptional changes in *ERF*, *MYB* and *WRKY* genes, alongside hormone signal transduction components under salt stress (Li et al., 2022).

In these systems, activation of stress-responsive transcription factors by melatonin correlates with enhanced expression of downstream functional genes involved in ROS detoxification, ion transport, osmolyte synthesis and cell wall remodeling, thereby contributing to improved root resilience to abiotic stresses. This regulatory mode provides a molecular genetics framework explaining how melatonin improves root vitality and stress resilience beyond direct ROS scavenging. Recent studies have showed that melatonin perception in plants is at least partially mediated by the plasma membrane receptor CAND2/PMTR1 (Bychkov et al., 2022). In *Arabidopsis*, CAND2/PMTR1 has been functionally validated as a melatonin receptor required for melatonin-induced stress tolerance and ROS homeostasis (Wang et al., 2021b), highlighting receptor-mediated signaling as a critical direction for future melatonin research.

## 6 Melatonin mediated root-microbe interactions

Root system architecture is closely linked to rhizosphere microbial communities, including mycorrhizal fungi and plant growth-promoting rhizobacteria (Ye et al., 2022). Emerging evidence suggests that melatonin may indirectly modulate root-microbe interactions by altering root exudation patterns, redox status, and hormone signaling (Madigan et al., 2019; Xia et al., 2022; Ye et al., 2022). These changes could influence microbial colonization, symbiotic efficiency, and nutrient acquisition under stress conditions. For example, exogenous melatonin altered rhizosphere microbial community composition in barley under drought stress, enriching certain bacterial taxa and modifying functional profiles of the microbiome in ways correlated with improved drought tolerance (Ye et al., 2022). Similarly, melatonin application increased arbuscular mycorrhizal (AM) colonization and enhanced nutrient uptake in legume seedlings under drought, indicating melatonin-AM synergism in root-microbe interactions (Xia et al., 2022).

## 7 Conclusions and future perspectives

Plant roots are essential for plant growth, stress perception, and environmental adaptation, and accumulating evidence establishes melatonin as a key integrative regulator of root system architecture (De Smet et al., 2012; Giehl et al., 2014). At appropriate concentrations, exogenous melatonin promotes primary, lateral, and adventitious root development, delays stress-induced root senescence, and enhances whole-plant performance under adverse conditions (Li et al., 2018; Hu et al., 2020; Gao et al., 2022). Mechanistically, melatonin functions in a concentration-dependent manner, coordinating multiple phytohormone pathways—particularly through synergistic interactions with auxin and antagonistic regulation of stress-associated hormones such as abscisic acid—while maintaining redox homeostasis via its antioxidant capacity (Arnao and Hernández-Ruiz, 2018; Bian et al., 2021). In parallel, melatonin modulates nutrient acquisition and metabolic reprogramming, underscoring its pleiotropic role in sustaining root growth under environmental stress (Huang et al., 2020; Shi et al., 2015).

From an applied perspective, melatonin represents a promising and environmentally friendly regulator for horticultural and agricultural production (Wu et al., 2021). Its capacity to stimulate adventitious root formation, improve root performance under flooding or nutrient deficiency, and enhance vegetative propagation highlights its practical value as a supplement or alternative to conventional chemical regulators (Zhang et al., 2013; Iqbal and Khan, 2022; Zeng et al., 2022b; Cano et al., 2024). Moreover, emerging evidence suggests that melatonin may indirectly influence root–microbe interactions by reshaping hormonal balance, redox status, and root physiology, although direct experimental validation remains limited (Madigan et al., 2019; Ye et al., 2022).

Despite rapid progress, key knowledge gaps remain. The identification and functional characterization of melatonin receptors in roots, as well as downstream signaling components, are critical for understanding melatonin perception and signaling specificity. In addition, post-transcriptional regulatory layers, including miRNA-mediated pathways and RNA modification, remain largely unexplored in the context of melatonin-regulated root development. Future studies integrating genetic mutants, cell-type-specific analyses, and multi-omics approaches will be essential to unravel the context-dependent roles of melatonin in root systems and to facilitate its rational application in sustainable crop production.
